# Fungal and Bacterial Microbiome Associated with the Rhizosphere of Native Plants from the Atacama Desert

**DOI:** 10.3390/microorganisms8020209

**Published:** 2020-02-04

**Authors:** Alejandra Fuentes, Héctor Herrera, Trevor C. Charles, Cesar Arriagada

**Affiliations:** 1 Laboratorio Biorremediación, Departamento de Ciencias Forestales, Facultad de Ciencias Agropecuarias y Forestales, Universidad de La Frontera, Francisco Salazar, Temuco 01145, Chile; alejandra.fuentes@ufrontera.cl (A.F.); hector.herrera@ufrontera.cl (H.H.); 2 Department of Biology, University of Waterloo, University Avenue West, Waterloo, ON N2L 3G1; Canada; trevor.charles@uwaterloo.ca

**Keywords:** abiotic stress, extreme environment, plant growth promotion

## Abstract

The rhizosphere microbiome is key in survival, development, and stress tolerance in plants. Salinity, drought, and extreme temperatures are frequent events in the Atacama Desert, considered the driest in the world. However, little information of the rhizosphere microbiome and its possible contribution to the adaptation and tolerance of plants that inhabit the desert is available. We used a high-throughput Illumina MiSeq sequencing approach to explore the composition, diversity, and functions of fungal and bacterial communities of the rhizosphere of *Baccharis scandens* and *Solanum chilense* native plants from the Atacama Desert. Our results showed that the fungal phyla Ascomycota and Basidiomycota and the bacterial phyla Actinobacteria and Proteobacteria were the dominant taxa in the rhizosphere of both plants. The linear discriminant analysis (LDA) effect size (LefSe) of the rhizosphere communities associated with *B. scandens* showed the genera *Penicillium* and *Arthrobacter* were the preferential taxa, whereas the genera *Oidiodendron* and *Nitrospirae* was the preferential taxa in *S. chilense.* Both plant showed similar diversity, richness, and abundance according to Shannon index, observed OTUs, and evenness. Our results indicate that there are no significant differences (*p* = 0.1) between the fungal and bacterial communities of both plants, however through LefSe, we find taxa associated with each plant species and the PCoA shows a separation between the samples of each species. This study provides knowledge to relate the assembly of the microbiome to the adaptability to drought stress in desert plants.

## 1. Introduction

The rhizosphere is the zone of soil around the roots and is influenced by root exudates regulating proliferation and the activity of several soil microorganisms [1]. Here, a large number of interactions between microorganisms and invertebrates occur, affecting the biogeochemical cycles and the growth and tolerance of plants against biotic or abiotic stress [2]. Furthermore, the biological and chemical activities of the rhizosphere are influenced by the metabolic compounds exuded by the roots, including organic acids, sugars, amino acids, small peptides, secondary metabolites, among others [3]. These organic compounds increase the growth of the microbiota and also act as chemical signals with different effects in the plants. The interaction between microorganisms and rhizosphere may vary according to the soil conditions resulting in beneficial, detrimental, or neutral effects to plants [4]. 

Rhizosphere microorganisms can promote plant growth by direct and indirect mechanisms. As for the direct mechanisms, the most relevant are induction of systemic acquired resistance (SAR) in the plant, and production of phytohormones such as auxin and gibberellins. On the other hand, indirect mechanisms include production of siderophores [5,6], production of low molecular weight organic acids [7,8], mineralization of organic matter making nutrients available for the plants, protection against phytopathogens, among others [9]. Under drought stress, changes in the structure of the microbial communities associated with the roots have been observed indicating a selective pressure determined by the plant on the rhizosphere microbiome [10]. 

On the other hand, microorganisms isolated from arid ecosystems are more effective as inducers of stress tolerance in plants growing under drought stress than microorganisms not adapted to this condition, demonstrating greater efficiency in plant growth promotion and tolerance to stress mainly by improvement in stomatal conductance, nutrition, and osmotic adjustment [11,12]. 

The Atacama Desert is located in northern Chile and is described as the driest and oldest in the world [13]. In addition to aridity, the Atacama Desert has other environmental factors such as strong thermal oscillation, low relative humidity levels, low water retention [14], high levels of ultraviolet radiation [15], extreme oligotrophic conditions, high salinity, and high levels of inorganic oxidants in soil [16]. 

Despite this, the Atacama Desert harbors a great diversity of vascular plants consisting of around 600 species, belonging to 304 genera and 81 families. The families best represented in vascular plants are the Asteraceae, Poaceas, Fabaceas, Malvaceas, and Solanaceae, which have adapted to poly-extreme environment through mechanisms that contribute to plant tolerance and development [17]. Additionally, the influence of soil microbial diversity on plants is a key factor in the plant adaptation to this arid ecosystem [18]. However, few studies have associated the rhizosphere microbiome of desert native plants with the induction of tolerance to abiotic stress.

For the more complete derivation of the structure and function of the rhizosphere microbiome, identifying all the microbes present and their functions is very difficult to achieve with traditional tools, because most are uncultured or unknown its genomic sequences [19]. Metagenomics analysis includes the study of the entire spectrum of microorganisms present in a given sample through the direct sequencing of microbial community DNA. This have had gained great relevance allowing description of complete genomes to obtain not only information about the richness and relative abundance of microbial populations or explore their biotechnological potential, but also to understand the composition of the microbial community and assign a role to the uncultured components of the microbiome [20]. The analysis of the diversity of microbial communities has revealed taxa that may be commonly associated with the rhizosphere of plants. This has helped to understand the differences between rhizosphere soil of different plant species or in different compartments inside the plant (rhizoplane, endosphere) allowing to understand the dynamics of the processes in the plant-microbiome interaction, as well as inferring if the predominance of certain groups is related to the functional structure of the microbiome [21]. 

*Baccaris scandens* and *Solanum chilense,* Asteraceae and Solanaceae family respectively grow in different habitats and are distributed in a wide range of altitudes (from sea level to high elevation in the Andes Mountains), and latitudinally extending into northern Chile [22] evidencing a high adaptability and tolerance to drought stress.

The aims of this study are: (i) to describe and compare fungal and bacterial communities of the rhizosphere of *Baccaris scandens* and *Solanum chilense*, and (ii) to identify potential microbial groups that could be involved in the tolerance of plants to these extreme environmental conditions.

## 2. Materials and Methods

### 2.1. Study Site and Sample Collection

The samples were collected during the summer (January 2018) in an area located between 19°18′ S and 69°25′ W in the Atacama Desert, northern Chile. This area is located between 2500 and 3200 m.a.s.l corresponding to the marginal desert of height. The average annual rainfall is of 36.7 mm [23] and the average temperature in summer (at the time of sample collection) reaches up to 21 °C during the day and between 5 and 8 °C during the night. In this area *B. scandens* and *S. chilense* have high coverage, whereby isolated plants were selected ensuring a minimum distance at least 3 m away from each sample plants and without any other plant species growing at a distance of 1 m.

Rhizophere soil (5 g) of three different individual plants of each of *B. scandens* and *S. chilense* was collected using sterile gloves and a clean spade to a depth of 5–10 cm. The rhizosphere soil was removed by gently shaking the plants, deposited in sterile polypropylene conical tubes and immediately stored on ice until their arrival at the laboratory.

The rhizosphere soil samples were subjected to chemical analysis. The available P content was determined by extraction with NaHCO_3_ at pH 8.5 [24]. The available K was determined according to Mingorance [25]. The organic matter (OM) was determined by the method described by Walkley and Black [26]. The pH was measured using 1:2.5 ratio of soil:deionized water. Exchangeable bases K, Na, Ca, and Mg were determined by extraction with 1 M NH_4_OAC [27]. The chemical analysis is shown in Table 1. 

### 2.2. DNA Extraction and High-Throughput Sequencing

Total DNA was extracted from 0.5 g of rhizosphere soil of *S. chilense* and *B. scandens,* using the PureLink^TM^ Microbiome DNA Purification Kit (Invitrogen, Carlsbad, CA, USA), following the manufacturer´s instructions. The quality and concentration of DNA were checked in a Qubit^®^ 2.0 Fluorometer (Thermo Fisher Scientific, Waltham, MA, USA).

DNA samples were sequenced by Macrogen, Inc. (Seoul, South Korea). The amplicon libraries were amplified using PCR according to the Illumina PCR Quantification Protocol Guide. The V3-V4 hypervariable region of the 16S rRNA gene was amplified using the primer set Bakt_341F (5′ CCTACGGGNGGCWGCAG 3′) and Bakt_805R (5′ GACTACHVGGGTATCTAATCC 3′) for bacteria [28], while ITS region of the 18S rRNA gene was amplified using the primer set ITS2_3F (5′ GCATCGATGAAGAACGCAGC 3′) and ITS2_4R (5′ TCCTCCGCTTATTGATATGC 3′) for fungi. To verify the size of PCR enriched fragments the size distribution was visualized on an Agilent Technologies 2100 Bioanalyser using a DNA 1000 chip. Concentration was between 52 and 93 ng/μL. After size verification, the libraries were sequenced using a 2 × 300-bp paired-end run [MiSeq Reagent Kit, v. 3 (MS-102-3001)] on a MiSeq (Illumina, San Diego, CA, USA) instrument according to instructions of the manufacturer (Illumina).

### 2.3. Sequence Analysis and Taxonomical Assignation

The quality of resulting raw reads was checked using FastQC v.0.11.5 [29] and taxonomic assignment was done using the software Quantitative Insights Into Microbial Ecology, QIIME2 [30]. Quality filter step was realized with DADA2 algorithm [31] as a QIIME2 plugin. Barcodes were removed and reads were truncated at length of 283 and 253 bp, for forward and reverse, respectively. In this primary step, paired-end reads were joined together and then a quality-aware correcting model for amplicon data that denoises, removes chimeras and residual PhiX reads, and dereplicates DNA reads was applied. Reads were then dereplicated and amplicon sequence variants (ASVs) were called. ASV generation was recently shown to outperform operational taxonomic unit (OTU) clustering, resulting in fewer spurious reads [32].

The taxonomic assignment of the ITS2-derived ASVs was performed using UNITE database version 7.2 (UNITE community, 2017). Because of the high variability in length on ITS2 region among species, which may lead to a non-optimal alignment resulting in a doubtful tree, phylogeny-related measurements of alpha (Faith PD) and beta (UniFrac) diversity were not calculated.

The taxonomic assignment of the16S-derived ASVs was performed using Greengenes version 13.8 [33] database as reference, where the specific region targeted by primers Bakt_341F and Bakt_805R was extracted to build the model. Phylogenetic relationships between ASVs was obtained by constructing a phylogenetic tree using FastTree algorithm [34] based on a masked alignment constructed with MAFFT [35].

### 2.4. Statistical Analysis

Alpha diversity measurements for diversity (Shannon’s diversity index and Faith’s Phylogenetic Diversity), richness (Observed OTU), and evenness (Pielou’s evenness) were calculated using QIIME2 and resulting values were compared between plant rhizospheres by Kruskall Wallis test. To visualize broad trends of overall bacterial and fungal communities between *B. scandens* and *S. chilense* rhizospheres, a principal coordinate analysis (PCoA) and a hierarchical clustering were constructed based on the Bray–Curtis dissimilarity matrix. Statistical determination of differences between plant rhizospheres were evaluated by permutational multivariate analysis of variance (PERMANOVA) [36] with 999 random permutations on a Bray–Curtis dissimilarity matrix. Statistical analysis and visualization were performed using R package *vegan* [37].

Linear discriminant analysis (LDA) effect size (LefSe) was used to identify biologically relevant features for any group using Kruskal–Wallis followed by a Wilcoxon rank-sum test for pairwise comparison, with a *p*-value of 0.05 as cut-off and a linear discriminant analysis score of 2.0 [38] and then *IndicSpecies,* was used to analyze the strength and statistical significance of the relationship between species occurrence and abundance with groups of sites [39,40].

## 3. Results

### 3.1. Sequencing Results and Quality Control

The ITS2 sequencing produced a total of 1,425,157 raw reads across 6 input libraries. After quality filtering, 931,011 ASVs were retained, ranging from 139,214 to 166,516, and an average of 155,168 ASVs (Table S1).

The 16S sequencing produced a total of 1,245,865 raw reads across 6 input libraries. After quality filtering, 275,581 ASVs were retained, ranging from 38,816 to 54,381, and an average of 45,930 ASVs (Table S2).

### 3.2. Taxonomic Composition of Fungal and Bacterial Communities in Rhizosphere Soil

**Figure 1:**
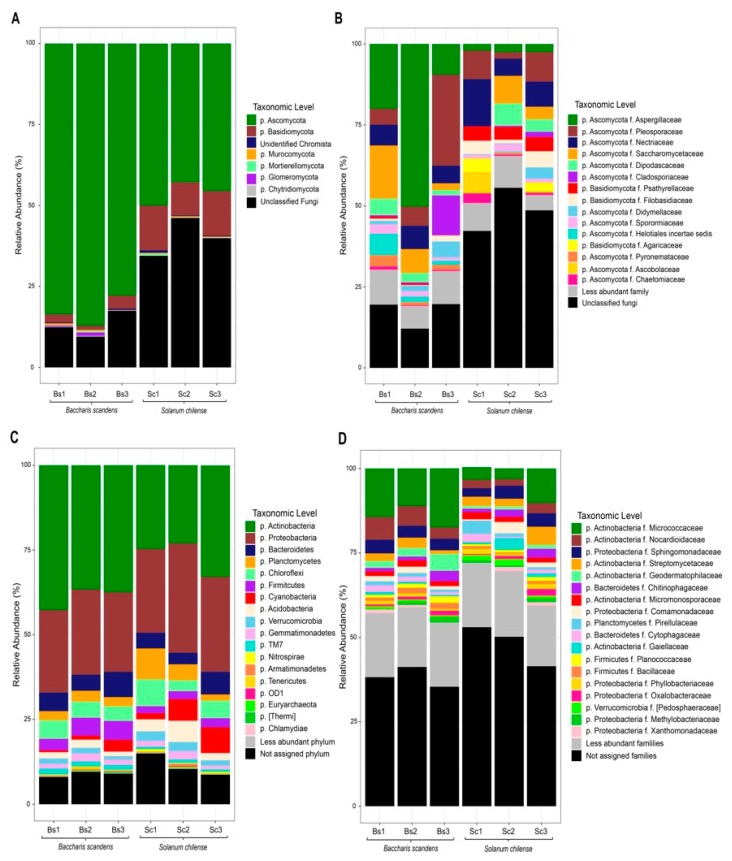
Taxonomic composition of the rhizosphere soil of *B. scandens* and *S. chilense,* growing naturally in the Atacama Desert. Average of relative abundance of fungal (**A**) and bacterial (**C**) phyla and fungal (**B**) and bacterial (**D**) families.

The relative abundances of members of the fungal community in rhizosphere of *B. scandens* and *S. chilense* are shown in Figure 1. At the phylum level (Figure 1A) Ascomycota taxa showed a clear dominance in *B*. *scandens* and *S. chilense*, with a mean relative abundance of 82.81 and 46.08%, respectively. The second most abundant phylum was Basidiomycota with 2.60 and 12.64% in *B*. *scandens* and *S. chilense* respectively. The remaining phyla have relative abundance smaller than 1%, composed of unclassified chromista (0.27% to 0.42%), Mucuromycota (0.30% to 0.26%), Mortierellomycota (0.24% to 0.23%), Chytridiomycota (0.15% to 0.18%), and the Glomeromycota that only was detected in rhizosphere soil of *B. scandens* (0.50%) (Table S3).

At family level (Figure 1B), rhizosphere soil of *B. scandens* showed a higher abundance of Aspergillaceae (26.57%), followed by Pleosporaceae (6.65%), Saccharomycetaceae (8.57%), Nectricaceae (6.34%), Cladosporaceae (4.33%), Dipodascaceae (3.26%), Heliotales (3.16%), Dydimellaceae (2.63%). In contrast, Nectricaceae (9.2%) was the dominant family in the rhizosphere of *S. chilense*, followed by the Pleosporaceae (12%), Psathyrellaceae (4.25%), Saccharomycetaceae (4.1%), Dipodascaceae (3.61%), Filobasidiaceae (3.4%), Agaricaceae (2.46%), and Aspergillaceae (2.34%) (Table S4).

The composition of rhizosphere bacterial community (Figure 1C) showed Actinobacteria as the most abundant phylum of *B. scandens* with a mean relative abundance of 38%, and Proteobacteria as the most abundant phylum of *S. chilense* with a mean relative abundance of 28.4%. The second most abundant phylum of *B. scandens* were Proteobacteria (24.4%) and Actinobacteria (26.9%) in *S. chilense*. The remaining abundance was composed of Bacteroidetes (5.89% to 4.93%), Planctomycetes (2.82 to 5.18%), Chloroflexi (4.88 to 5.46%), Cyanobacteria (1.75 to 5.27%), Acidobacteria (1.85 to 3.98%), Verrucomicrobia (1.47 to 2.41%), Gemmatimonadetes (1.74 to 1.73%), and the 1% of less abundant phyla Nitrospirae (0.17 to 0.49%), Armatimonadetes (0.11 to 0.31%), Tenericutes (0.33 to 0%) (Table S5).

At the family level (Figure 1D), *B. scandens* rhizosphere showed more relative abundances of Micrococcaceae (14.3%), Nocardioidaceae (5.29%), Sphingomonadaceae (3.7%), Geodermatophilaceae (3.23%), and Streptomycetaceae (2.06%). In *S. chilense* rhizosphere showed higher relative abundances of Micrococcaceae (5.68%), Sphingomonadaceae (3.46%), Streptomycetaceae (3.4%), Rhodospirillaceae (3.06%), and Chitinophagaceae (1.93%) (Table S6).

### 3.3. Structure of Fungal and Bacterial Communities in the Rhizosphere

**Figure 2:**
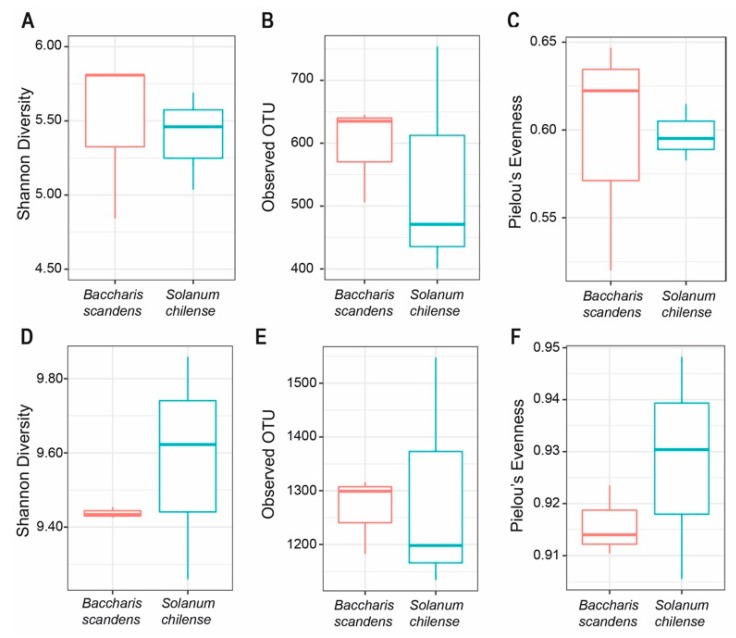
Alpha diversity measurements distribution of bacterial and fungal communities in rhizosphere soil of *B. scandens* and *S. chilense*. (**A**) Shannon index of fungi. (**B**) Richness of fungi. (**C**) Evenness of fungi. (**D**) Shannon index of bacteria. (**E**) Richness of bacteria. (**F**) Evenness of bacteria.

The structure of microbial communities detected in the rhizosphere of both plants is shown in Figure 2. Rhizosphere fungal communities between *B. scandens* and *S. chilense* did not show significant differences according to Wilcoxon Rank Sum test applied to alpha diversity measurements (Shannon index; *p* = 0.7), richness (observed OTU, *p* = 0.7) and evenness (Pierlou’s evenness, *p* = 0.7) (Figure 2A–C) (Table S7). 

With respect to rhizosphere bacterial communities, Kruskal–Wallis analysis did not show significant differences in values of diversity (Shannon index; *p* = 0.7), richness (observed OTU, *p* = 1.0), and evenness (Pierlou’s evenness, *p* = 0.7) when the rhizosphere of *B. scandens* and *S. chilense* were compared (Figure 2D–F) (Table S8).

**Figure 3:**
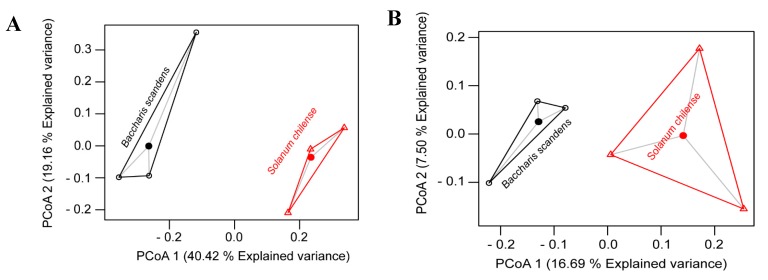
Principal coordinate analysis (PCoA) of fungal (**A**) and (**B**) bacterial communities from rhizosphere of *B. scandens* and *S. chilense.*

To observe the similarities and dissimilarities among soil rhizospheres of both plants, principal co-ordinates analysis (PCoA) and clustering analysis were performed for fungi (Figure 3A) and bacteria (Figure 3B). These plots showed a clear grouping between rhizosphere microorganisms of *B. scandens* and *S. chilense* plants, however PERMANOVA test on Bray–Curtis dissimilarity, showed no significant differences between *B. scandens* and *S. chilense* rhizosphere neither in fungal (*p*= 0.1000, *F* = 3.8072, *r*^2^ = 0.4877) and bacterial communities (*p*= 0.1000, *F* = 2.3903, *r*^2^ = 0.3741).

### 3.4. Analysis of Specific Taxa of Fungal and Bacterial in the Rhizosphere

A LefSe analysis was used to compare microbial communities and identify specific microorganisms at phylum and genus level of rhizosphere soil.

**Figure 4:**
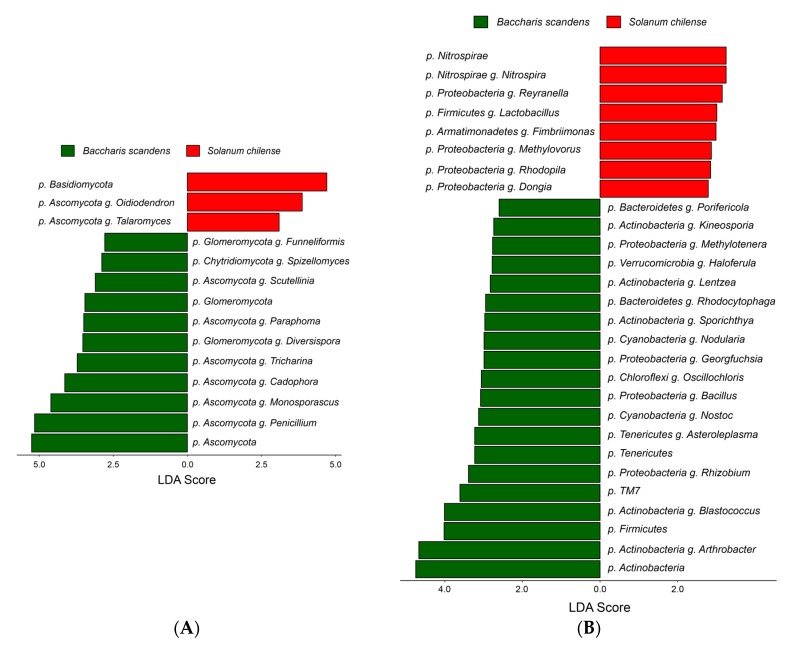
Linear discriminate analysis (LDA) of effect size (LEfSe) to identify preferential taxa at the phylum (p) and genus (g) levels in rhizosphere fungi (**A**) and bacteria (**B**) of *B. scandens* and *S. chilense*. Only taxa with an LDA score > 2.0 are shown.

At the phylum level in fungi (Figure 4A), the relative abundance of OTUs from Glomeromycota and Ascomycota were significantly higher in *B. scandens*, with LDA score of 5.26 and 3.46 respectively. Meanwhile Basidiomycota phylum had a higher relative abundance in *S. chilense* with LDA score of 4.71. At the genus level, *Penicillium* was the most differential taxon in *B. scandens* (LDA score = 5.16), followed by *Cadophora*, *Tricharina*, *Paraphoma*, *Spizellomyces*, *Funneliformis,* and *Monosporascus* (Table S9). 

In bacteria at the phylum level (Figure 4B), Actinobacteria was the most differential taxon in *B. scandens* rhizosphere with LDA score of 4.75, followed by Firmicutes (LDA score = 4.02), TM7 (LDA score = 3.61), and Tenericutes (LDA score = 3.23). The phylum Nitrospirae was significantly more abundant in *S. chilense* rhizosphere. 

At genus level 16 features were significantly more abundant in the *B. scandens* rhizosphere, where *Arthrobacter* and *Blastococcus* showed high abundance (LDA score = 4.67 and 4.01 respectively), followed by *Rhizobium*, *Asteroleplasma*, *Nostoc*, *Bacillus*, *Oscillochloris*, *Nodularia*, *Georgfuchsia*, *Lentzea*, *Rhodocytophaga*, *Sporichthya*, *Haloferula*, *Methylotenera*, *Kineosporea*, *Porifericola*. Meanwhile seven features were significantly more abundant in the rhizosphere of *S. chilense, Nitrospira* and *Reyranella* being the most differential taxa (LDA score = 3.25 and 3.15 respectively), followed by *Lactobacillus*, *Fibriiomonas*, *Rhodopila*, *Methylovorus*, and *Dongia.* IndicSpecies analysis showed that none of the taxa in fungi or bacteria were associated to the *B. scandens* or *S. chilense* (Table S10).

## 4. Discussion

Plants that inhabit the Atacama Desert have been studied for their high tolerance to drought, salinity, radiation, and extreme temperatures. In particular, *S. chilense*, given its high tolerance to hydric deficit, has been used as a model plant in physiological and molecular studies [41,42]. In our study, we have focused on the rhizosphere, in order to explore a possible role of microorganisms in the induction of tolerance to the hydric deficit characteristic of arid zones. We have described the fungal and bacterial communities of the rhizosphere of two native plants from the Atacama Desert and have identified groups present in the rhizosphere microbiome and that could be involved in the establishment and development of the plant under the restrictive conditions in arid ecosystems.

Our results showed that both plants harbor *Ascomycota* as the dominant phyla followed *Basidiomycota*. The predominance of these phylum has also been reported in studies where a variety of cultivable fungi from the bulk soil [43], decaying wood from ancient saltpeters [44], rocks [45] has been isolated. Despite the ecological roles of the fungi in the in ecosystems, its distribution and diversity in the plant interaction (rhizosphere microbiome) has been poorly described in Atacama Desert. González-Teuber et al. [46] isolated root-endophytic fungi from *Chenopodium quinoa* a pseudo-cereal well-adapted to the climatic conditions of the Atacama Desert where *Ascomycota* was the dominant phylum.

Similar to our study, in metagenomics analysis of rhizosphere fungal communities in arid and semi-arid ecosystems, Suleiman et al. [47] and Vargas-Gastélum et al. [48] also showed a high relative abundance of Ascomycota and Basidiomycota phyla in the rhizosphere of *Vachellia pachyceras* and plants from Valle de Las Palmas, respectively. While the composition of fungal communities in the bulk also show a high abundance of these phyla [49,50,51], so their presence would be associated with type of soil and climate which is decisive for the rhizospheric microbiome assembly process.

On the other hand Ascomycota is known to include “dark septate endophytes” (DSEs) and has been observed in roots of plants that grow in stressful and nutrient-limited [52] and have shown a positive effect on plant growth [53,54] probably because of nitrogen mineralization and pathogen protection [55].

At family level, we found difference between rhizosphere fungal communities of *B. scandens* and *S. chilense,* Asperguillaceae and Nectriaceae being the principal fungal family, respectively. Members of these families have been reported as inducers of tolerance to water deficit in different plant species, through the production of metabolites such as (Z)-N(4-hydroxystyryl) formamide (NFA) [56] and the phytohormones production [57]. According to LeFSe analysis (LDA > 2) we showed preferential taxa in each plant, indicating a direct effect of the plant species on the rhizosphere microbial community. In *B. scandens* at genus level we observed a high abundance the OTUs assigned to the genera *Penicillium*, a genus characterized by promoting plant growth by solubilization of minerals, phytohormones production, and biological control against phytopathogens [58]. In our study, we found the phylum Glomeromycota significantly enriched in *B. scandens*; it includes arbuscular mycorrhizal fungi (AMFs) [59]. AMFs form specialized structures within the root cortex known as arbuscules, where the transfer of nutrients between the two symbionts occurs. In this symbiosis, the fungus supplies to the plant inorganic compounds and the plant contributes to the heterotrophic fungus with organic compounds (photosynthates) [60]. The OTUs were assigned to the genera *Funneliformis* and *Diversispora*. These genera have been found in high abundance in arid and saline soils and recently in the rhizosphere of *Larrea tridentata,* a perennial plant of Chihuahuan Desert [61] and in *Hedysarum scoparium* in desert areas of northern China [62]. Arbuscular mycorrhizal symbiosis directly influences the uptake and transfer of water through the hyphae of the fungus [63,64], and also improve the osmoregulation mechanisms in the host plant to tolerate drought and salt stress, thus increasing compatible osmolytes, such as proline, betaine, polyamines, sugars, organic acids, amino acids, and trehalose [65,66]. Furthermore, AMF increases gas exchange and water use efficiency [67], increases photosynthetic efficiency, improves the antioxidant enzyme response, and induces the accumulation of antioxidant molecules such as glutathione and decrease lipid peroxidation of membranes [67,68,69]. The root hydraulic conductivity is also affected by mycorrhizal colonization, which is evidenced in proteins involved in water transport [70]. In our study, the presence of this phylum in the rhizosphere of the plants is directly related to the greater tolerance of *B. scandens* to stress conditions due to drought in the Atacama Desert.

The bacterial community has been widely studied in different niches of the Atacama Desert such as rocks, surface of soil, geothermal field, among others [71] where Actinobacteria and Proteobacteria phyla are frequently found. In addition, we found these dominant phyla in the rhizosphere of *B. scandens* and *S. chilense* and have been identified in the rhizosphere of *Atriplex* sp. and *Stipa* sp. also native of the Atacama Desert [72] and in other arid ecosystems, *Tribulus terrestris*, *Zygophyllum simplex*, *Panicum turgidum,* and *Euphorbia granulata* of Saudi Arabia Desert [73]. Also, recent studies of endophytic bacterial communities in *Distichilis spicata* and *Pluschea absinthioides* have identified Proteobacteria as a higher dominant phylum [74].

The presence of Microccaceae family in soils of the Atacama Desert had already been previously described by Idris et al. [75] in soils. In our study, this family was the most abundant in the rhizosphere of both plants. With respect to the possible role in the plant tolerance to abiotic stress, several genus of this family have been described for their ability to control of plant pathogen [76], phosphate solubilization, phytohormone production, siderophores production [77,78].

Other families such as Nocardioidaceae, Sphingomonadaceae, Streptomycetaceae, were found in both plants, with different relative abundances. Nocardioidaceae and Streptomycetaceae families have been previously reported in rhizosphere [74,79] and bulk soil [75] in native plants of the Atacama Desert Altiplano. With respect to the family Sphingomonadaceae different strains of Sphingomonas spp. have shown a positive effect on plant growth promotion [80] even under heavy metal [81] and drought stress [82]. LeFSe analysis showed the genus *Arthrobacter* was present in both rhizosphere plant but significantly enriched in rhizosphere of *B. scandens.* This genus had been previously isolated from Atacama Desert [71], and have been previously reported as a basic constituents of rhizosphere and have positive effects on plants as a phosphate solubilizers and antagonist of plant parasitic nematodes [83]. In the rhizosphere of *S. chilense* the phylum Nitrospirae was the most significantly enriched with genus *Nitrospira* essential in the process of nitrification and transformation of nitrite to nitrate [84].

*B. scandens* and *S. chilense* are plants adapted to the extreme weather conditions of the Atacama Desert. We found that the Ascomycota and Basidiomycota fungal phyla and Actinobacteria and Proteobacteria bacterial phyla were the dominant taxa in both rhizospheres but with differences in relative abundance at the family level. In this sense, several of the families found are recognized for their role in stress protection and plant growth promotion.

As for the effect of the plant species on the structure and assembly of the root microbiome, with our sampling it was not possible to reach a significance level that indicate differences between communities of each plant species, however these communities are clearly separated on PCoA, therefore it suggests the need to counter with greater statistical power to clearly detect this difference.

The extreme conditions of the Atacama Desert generate a selective pressure of microorganisms in the soil and the plant recruit specific families of fungi and bacteria that can have a positive effect on plant growth, tolerance, and adaptation to different abiotic stress (drought, high salinity, temperature, radiation, among others).

Understanding the rhizosphere microbiome composition and assemblages in the driest place in the world is relevant for the development of plant protection strategies under a climate change scenario and can contribute to improve the tolerance of plant to drought stress.

## Figures and Tables

**Table 1:** Chemical analysis of rhizosphere soil from *B. scandens* and *S. chilense* growing naturally in Atacama Desert.

Samples	*B. scandens*	*S. chilense*
P_Olsen_ (mg kg^−1^)	5	9
K (mg kg^−1^)	328	411
Organic matter (%)	0.91	1.21
pH _H2O_	7.2	6.32
K (cmol _(+)_kg^−1^)	0.84	1.05
Na (cmol _(+)_ kg^−1^)	1.26	0.55
Ca (cmol _(+)_ kg^−1^)	9.03	7.87
Mg (cmol _(+)_ kg^−1^)	1.36	2.04
CEC * (cmol _(+)_ kg^−1^)	12.51	11.54

* CEC = catión Exchange capacity = Σ(K, Ca, Mg y Mg).

## Supplementary Materials

The following are available online at https://www.mdpi.com/2076-2607/8/2/209/s1.

## References

[1] Lynch J.M., de Leij F. (2012). Rhizosphere. eLS.

[2] Philippot L., Raaijmakers J.M., Lemanceau P., van der Putten W.H. (2013). Going back to the roots: The microbial ecology of the rhizosphere. Nat. Rev. Microbiol..

[3] Dakora F., Phillips D. (2002). Root exudates as mediators of mineral acquisition in low-nutrient environments. Plant Soil.

[4] Singh A.K., Varaprasad K.S. (2008). Criteria for identification and assessment of agro-biodiversity heritage sites: Evolving sustainable agriculture. Curr. Sci..

[5] Adesemoye A., Kloepper J. (2009). Plant–microbes interactions in enhanced fertilizer-use efficiency. Appl. Microbiol. Biotechnol..

[6] Ramos-Solano B., Lucas García J.A., Garcia-Villaraco A., Algar E., Garcia-Cristobal J., Gutierrez Mañero F.J. (2010). Siderophore and chitinase producing isolates from the rhizosphere of Nicotiana glauca Graham enhance growth and induce systemic resistance in Solanum lycopersicum L.. Plant Soil.

[7] Avis T.J., Gravel V., Antoun H., Tweddell R.J. (2008). Multifaceted beneficial effects of rhizosphere microorganisms on plant health and productivity. Soil Biol. Biochem..

[8] Vassilev N., Vassileva M., Nikolaeva I. (2006). Simultaneous P-solubilizing and biocontrol activity of microorganisms: Potentials and future trends. Appl. Microbiol. Biotechnol..

[9] Olanrewaju O.S., Glick B.R., Babalola O.O. (2017). Mechanisms of action of plant growth promoting bacteria. World J. Microbiol. Biotechnol..

[10] Marasco R., Rolli E., Ettoumi B., Vigani G., Mapelli F., Borin S., Abou-Hadid A.F., El-Behairy U.A., Sorlini C., Cherif A. (2012). A Drought Resistance-Promoting Microbiome Is Selected by Root System under Desert Farming. PLoS ONE.

[11] Armada E., Roldán A., Azcon R. (2014). Differential Activity of Autochthonous Bacteria in Controlling Drought Stress in Native Lavandula and Salvia Plants Species Under Drought Conditions in Natural Arid Soil. Microb. Ecol..

[12] Ortiz N., Armada E., Duque E., Roldán A., Azcón R. (2015). Contribution of arbuscular mycorrhizal fungi and/or bacteria to enhancing plant drought tolerance under natural soil conditions: Effectiveness of autochthonous or allochthonous strains. J. Plant Physiol..

[13] Clarke J.D.A. (2006). Antiquity of aridity in the Chilean Atacama Desert. Geomorphology.

[14] Dose K., Bieger-Dose A., Ernst B., Feister U., Gómez-Silva B., Klein A., Risi S., Stridde C. (2001). Survival of microorganisms under the extreme conditions of the Atacama Desert. Orig. Life Evol. Biosph..

[15] Cordero R.R., Seckmeyer G., Damiani A., Riechelmann S., Rayas J., Labbe F., Laroze D. (2014). The world’s highest levels of surface UV. Photochem. Photobiol. Sci..

[16] Navarro-González R., Rainey F.A., Molina P., Bagaley D.R., Hollen B.J., de la Rosa J., Small A.M., Quinn R.C., Grunthaner F.J., Cáceres L. (2003). Mars-like soils in the Atacama Desert, Chile, and the dry limit of microbial life. Science.

[17] Hernández Palma J., Estades Marfán C., Faúndez Yancas L., Herreros de Lartundo J. (2014). Biodiversidad Terrestre de la Región de Arica y Parinacota.

[18] de Zelicourt A., Al-Yousif M., Hirt H. (2013). Rhizosphere Microbes as Essential Partners for Plant Stress Tolerance. Mol. Plant.

[19] Aguiar-Pulido V., Huang W., Suarez-Ulloa V., Cickovski T., Mathee K., Narasimhan G. (2016). Metagenomics, Metatranscriptomics, and Metabolomics Approaches for Microbiome Analysis. Evol. Bioinform. Online.

[20] Schlaeppi K., Bulgarelli D. (2014). The Plant Microbiome at Work. Mol. Plant-Microbe Interact..

[21] Mendes R., Garbeva P., Raaijmakers J.M. (2013). The rhizosphere microbiome: Significance of plant beneficial, plant pathogenic, and human pathogenic microorganisms. FEMS Microbiol. Rev..

[22] Warnock S.J. (1991). Natural habitats of Lycopersicon species. HortScience.

[23] Bustamante A.M. Caracterización de Humedales Altoandinos para una Gestión Sustentable de las Actividades Productivas del Sector Norte del país. Centro de Información de Recursos Naturales. http://bibliotecadigital.ciren.cl/handle/123456789/6295.

[24] Olsen S., Sommers L. (1982). Phosphorus. Methods Soil Analysis Part 2.

[25] Mingorance M. (2002). Focused microwave-assisted digestion of vegetal materials for the determination of essential mineral nutrients. Anal. Bioanal. Chem..

[26] Walkley A., Black I.A. (1934). An Examination of the Degtjareff Method for Determining Soil Organic Matter, and a Proposed Modification of the Chromic Acid Titration Method. Soil Sci..

[27] Warncke D., Brown J.R., Brown J.R. (1998). Potassium and Other Basic Cations. Recommended Chemical Soil Test Procedures for the North Central Region.

[28] Herlemann D.P., Labrenz M., Jürgens K., Bertilsson S., Waniek J.J., Andersson A.F. (2011). Transitions in bacterial communities along the 2000 km salinity gradient of the Baltic Sea. ISME J..

[29] Andrews S. FastQC. A Quality Control Tool for High Throughput Sequence Data. http://www.bioinformatics.babraham.ac.uk/projects/fastqc/.

[30] Kuczynski J., Stombaugh J., Walters W.A., González A., Caporaso J.G., Knight R. (2011). Using QIIME to analyze 16S rRNA gene sequences from microbial communities. Curr. Protoc. Bioinform..

[31] Callahan B.J., McMurdie P.J., Rosen M.J., Han A.W., Johnson A.J.A., Holmes S.P. (2016). DADA2: High-resolution sample inference from Illumina amplicon data. Nature Methods.

[32] Callahan B.J., McMurdie P.J., Holmes S.P. (2017). Exact sequence variants should replace operational taxonomic units in marker-gene data analysis. ISME J..

[33] McDonald D., Price M.N., Goodrich J., Nawrocki E.P., DeSantis T.Z., Probst A., Andersen G.L., Knight R., Hugenholtz P. (2012). An improved Greengenes taxonomy with explicit ranks for ecological and evolutionary analyses of bacteria and archaea. ISME J..

[34] Price M.N., Dehal P.S., Arkin A.P. (2010). FastTree 2—Approximately Maximum-Likelihood Trees for Large Alignments. PLoS ONE.

[35] Katoh K., Standley D.M. (2013). MAFFT multiple sequence alignment software version 7: Improvements in performance and usability. Mol. Biol. Evol..

[36] Anderson M.J. (2001). A new method for non-parametric multivariate analysis of variance. Austral Ecol..

[37] Oksanen J., Blanchet G., Friendly M., Kindt R., Legendre P., McGlinn D., Minchin P., O’Hara R., Simpson G., Solymos P. (2017). Vegan: Community Ecology Package. R Package Version 2.5-2. https://cran.r-project.org/package=vegan.

[38] Segata N., Izard J., Waldron L., Gevers D., Miropolsky L., Garrett W.S., Huttenhower C. (2011). Metagenomic biomarker discovery and explanation. Genome Biol..

[39] De Cáceres M., Jansen F. (2016). Indicspecies: Relationship between Species and Groups of Site. R Package Version 1.7.6. https://cran.r-project.org/package=indicspecies.

[40] Cáceres M.D., Legendre P. (2009). Associations between species and groups of sites: Indices and statistical inference. Ecology.

[41] Tapia G., Méndez J., Inostroza L. (2016). Different combinations of morpho-physiological traits are responsible for tolerance to drought in wild tomatoes Solanum chilense and Solanum peruvianum. Plant. Biol..

[42] Böndel K.B., Nosenko T., Stephan W. (2018). Signatures of natural selection in abiotic stress-responsive genes of Solanum chilense. R. Soc. Open Sci..

[43] Conley C.A., Ishkhanova G., McKay C.P., Cullings K. (2006). A Preliminary Survey of Non-Lichenized Fungi Cultured from the Hyperarid Atacama Desert of Chile. Astrobiology.

[44] Ortiz R., Navarrete H., Navarrete J., Párraga M., Carrasco I., Vega E.d.l., Ortiz M., Herrera P., Blanchette R.A. (2014). Deterioration, decay and identification of fungi isolated from wooden structures at the Humberstone and Santa Laura saltpeter works: A world heritage site in Chile. Int. Biodeterior. Biodegrad..

[45] Gonçalves V.N., Cantrell C.L., Wedge D.E., Ferreira M.C., Soares M.A., Jacob M.R., Oliveira F.S., Galante D., Rodrigues F., Alves T.M.A. (2016). Fungi associated with rocks of the Atacama Desert: Taxonomy, distribution, diversity, ecology and bioprospection for bioactive compounds. Environ. Microbiol..

[46] González-Teuber M., Vilo C., Bascuñán-Godoy L. (2017). Molecular characterization of endophytic fungi associated with the roots of Chenopodium quinoa inhabiting the Atacama Desert, Chile. Genom. Data.

[47] Suleiman M.K., Dixon K., Commander L., Nevill P., Quoreshi A.M., Bhat N.R., Manuvel A.J., Sivadasan M.T. (2019). Assessment of the Diversity of Fungal Community Composition Associated with Vachellia pachyceras and Its Rhizosphere Soil from Kuwait Desert. Front. Microbiol..

[48] Vargas-Gastélum L., Romero-Olivares A.L., Escalante A.E., Rocha-Olivares A., Brizuela C., Riquelme M. (2015). Impact of seasonal changes on fungal diversity of a semi-arid ecosystem revealed by 454 pyrosequencing. FEMS Microbiol. Ecol..

[49] Murgia M., Fiamma M., Barac A., Deligios M., Mazzarello V., Paglietti B., Cappuccinelli P., Al-Qahtani A., Squartini A., Rubino S. (2019). Biodiversity of fungi in hot desert sands. Microbiol. Open.

[50] Bates S.T., Garcia-Pichel F. (2009). A culture-independent study of free-living fungi in biological soil crusts of the Colorado Plateau: Their diversity and relative contribution to microbial biomass. Environ. Microbiol..

[51] Abed R.M.M., Al-Sadi A.M., Al-Shehi M., Al-Hinai S., Robinson M.D. (2013). Diversity of free-living and lichenized fungal communities in biological soil crusts of the Sultanate of Oman and their role in improving soil properties. Soil Biol. Biochem..

[52] Rothen C., Miranda V., Aranda-Rickert A., Fracchia S., Rodríguez M.A. (2017). Characterization of dark septate endophyte fungi associated with cultivated soybean at two growth stages. Appl. Soil Ecol..

[53] Newsham K.K. (2011). A meta-analysis of plant responses to dark septate root endophytes. New Phytol..

[54] Vergara C., Araujo K.E.C., Alves L.S., Souza S.R.D., Santos L.A., Santa-Catarina C., Silva K.D., Pereira G.M.D., Xavier G.R., Zilli J.É. (2018). Contribution of dark septate fungi to the nutrient uptake and growth of rice plants. Braz. J. Microbiol..

[55] Narisawa K., Surono (2018). The inhibitory role of dark septate endophytic fungus Phialocephala fortinii against Fusarium disease on the Asparagus officinalis growth in organic source conditions. Biol. Control..

[56] Qin W., Liu C., Jiang W., Xue Y., Wang G., Liu S. (2019). A coumarin analogue NFA from endophytic Aspergillus fumigatus improves drought resistance in rice as an antioxidant. BMC Microbiol..

[57] Hung R., Lee Rutgers S., Gupta V.K. (2016). Chapter 17—Applications of Aspergillus in Plant Growth Promotion. New Future Developments Microbial Biotechnology and Bioengineering.

[58] Altaf M.M., Imran M., Abulreesh H.H., Khan M.S.A., Ahmad I., Gupta V.K., Rodriguez-Couto S. (2018). Chapter 15—Diversity and Applications of Penicillium spp. in Plant-Growth Promotion. New and Future Developments in Microbial Biotechnology and Bioengineering.

[59] Schüβler A., Schwarzott D., Walker C. (2001). A new fungal phylum, the Glomeromycota: Phylogeny and evolution* *Dedicated to Manfred Kluge (Technische Universität Darmstadt) on the occasion of his retirement. Mycol. Res..

[60] Smith S.E., Read D., Smith S.E., Read D. (2008). The symbionts forming arbuscular mycorrhizas. Mycorrhizal Symbiosis.

[61] Hernández-Zamudio G., Sáenz-Mata J., Moreno-Reséndez A., Castañeda-Gaytán G., Ogaz A., Carballar-Hernández S., Hernández-Cuevas L. (2018). Dinámica de la diversidad temporal de los hongos micorrícicos arbusculares de Larrea tridentata (Sesse & Mocino ex DC) Coville en un ecosistema semiárido. Rev. Argent. Microbiol..

[62] Qiang W., He X., Wang J., Zhao L. (2019). Temporal and spatial variation of arbuscular mycorrhizal fungi under the canopy of Hedysarum scoparium in the northern desert, China. Appl. Soil Ecol..

[63] Ruiz-Lozano J.M., Azcón R. (1995). Hyphal contribution to water uptake in mycorrhizal plants as affected by the fungal species and water status. Physiol. Plant..

[64] Aroca R., Porcel R., Ruiz-Lozano J.M. (2012). Regulation of root water uptake under abiotic stress conditions. J. Exp. Bot..

[65] Aroca R., Porcel R., Ruiz-Lozano J.M. (2007). How does arbuscular mycorrhizal symbiosis regulate root hydraulic properties and plasma membrane aquaporins in Phaseolus vulgaris under drought, cold or salinity stresses?. New Phytol..

[66] Aggarwal A., Kadian N., Neetu K., Tanwar A., Gupta K. (2012). Arbuscular mycorrhizal symbiosis and alleviation of salinity stress. J. Appl. Nat. Sci..

[67] Ruiz-Sánchez M., Aroca R., Muñoz Y., Polón R., Ruiz-Lozano J.M. (2010). The arbuscular mycorrhizal symbiosis enhances the photosynthetic efficiency and the antioxidative response of rice plants subjected to drought stress. J. Plant. Physiol..

[68] Bompadre M.J., Silvani V.A., Bidondo L.F., Ríos de Molina M.D.C., Colombo R.P., Pardo A.G., Godeas A.M. (2014). Arbuscular mycorrhizal fungi alleviate oxidative stress in pomegranate plants growing under different irrigation conditions. Botany.

[69] Asrar A.-W.A., Elhindi K.M. (2011). Alleviation of drought stress of marigold (Tagetes erecta) plants by using arbuscular mycorrhizal fungi. Saudi J. Biol. Sci..

[70] Calvo-Polanco M., Sánchez-Castro I., Cantos M., García J.L., Azcón R., Ruiz-Lozano J.M., Beuzón C.R., Aroca R. (2016). Effects of different arbuscular mycorrhizal fungal backgrounds and soils on olive plants growth and water relation properties under well-watered and drought conditions. Plant Cell Environ..

[71] Azua-Bustos A., Urrejola C., Vicuña R. (2012). Life at the dry edge: Microorganisms of the Atacama Desert. FEBS Lett..

[72] Jorquera M.A., Maruyama F., Ogram A.V., Navarrete O.U., Lagos L.M., Inostroza N.G., Acuña J.J., Rilling J.I., de La Luz Mora M. (2016). Rhizobacterial Community Structures Associated with Native Plants Grown in Chilean Extreme Environments. Microb. Ecol..

[73] Eida A.A., Ziegler M., Lafi F.F., Michell C.T., Voolstra C.R., Hirt H., Saad M.M. (2018). Desert plant bacteria reveal host influence and beneficial plant growth properties. PLoS ONE.

[74] Zhang Q., Acuña J.J., Inostroza N.G., Mora M.L., Radic S., Sadowsky M.J., Jorquera M.A. (2019). Endophytic Bacterial Communities Associated with Roots and Leaves of Plants Growing in Chilean Extreme Environments. Sci. Rep..

[75] Idris H., Goodfellow M., Sanderson R., Asenjo J.A., Bull A.T. (2017). Actinobacterial Rare Biospheres and Dark Matter Revealed in Habitats of the Chilean Atacama Desert. Sci. Rep..

[76] Bano A., Muqarab R. (2017). Plant defence induced by PGPR against Spodoptera litura in tomato (Solanum lycopersicum L.). Plant Biol..

[77] Adhikari M., Yadav D.R., Kim S.W., Um Y.H., Kim H.S., Lee S.C., Song J.Y., Kim H.G., Lee Y.S. (2017). Biological control of bacterial fruit blotch of watermelon pathogen (Acidovorax citrulli) with rhizosphere associated bacteria. Plant Pathol. J..

[78] Dastager S.G., Deepa C.K., Pandey A. (2010). Isolation and characterization of novel plant growth promoting *Micrococcus* sp. NII-0909 and its interaction with cowpea. Plant. Physiol. Biochem..

[79] Fernández-Gómez B., Maldonado J., Mandakovic D., Gaete A., Gutiérrez R.A., Maass A., Cambiazo V., González M. (2019). Bacterial communities associated to Chilean altiplanic native plants from the Andean grasslands soils. Sci. Rep..

[80] Khan A.L., Waqas M., Kang S.-M., Al-Harrasi A., Hussain J., Al-Rawahi A., Al-Khiziri S., Ullah I., Ali L., Jung H.-Y. (2014). Bacterial endophyte Sphingomonas sp. LK11 produces gibberellins and IAA and promotes tomato plant growth. J. Microbiol..

[81] Pan F., Meng Q., Wang Q., Luo S., Chen B., Khan K.Y., Yang X., Feng Y. (2016). Endophytic bacterium Sphingomonas SaMR12 promotes cadmium accumulation by increasing glutathione biosynthesis in Sedum alfredii Hance. Chemosphere.

[82] Luo Y., Wang F., Huang Y., Zhou M., Gao J., Yan T., Sheng H., An L. (2019). Sphingomonas sp. Cra20 Increases Plant Growth Rate and Alters Rhizosphere Microbial Community Structure of Arabidopsis thaliana Under Drought Stress. Front. Microbiol..

[83] Mhatre P.H., Karthik C., Kadirvelu K., Divya K.L., Venkatasalam E.P., Srinivasan S., Ramkumar G., Saranya C., Shanmuganathan R. (2019). Plant growth promoting rhizobacteria (PGPR): A potential alternative tool for nematodes bio-control. Biocatal. Agric. Biotechnol..

[84] Daims H., Wagner M. (2018). Nitrospira. Trends Microbiol..

