# Supplementary material for: Fungal and Bacterial Microbiome Associated with the Rhizosphere of Native Plants from the Atacama Desert
Source: Microorganisms. 2020 Feb 4;8(2):209. doi: 10.3390/microorganisms8020209 (PMC7074712; doi:10.3390/microorganisms8020209)
Supplement: Supplementary file 1 [file microorganisms-08-00209-s001.pdf]

Supplementary table S1. Trimming summary ITS2, showing number of initial reads, reads that passes quality control, number of sequences after merging them by their 3' and resulting non-chimeric ASV.

SampleID	Input	Denoised	Merged	Non-chimeric
Bs1	237439	197038	180342	160835
Bs2	237572	191069	179966	163382
Bs3	226647	194003	185833	155899
Sc1	219013	179566	174092	139214
Sc2	235699	188823	181094	145165
Sc3	268787	221316	215965	166516

Supplementary table S2. Trimming summary 16S, showing number of initial reads, reads that passes quality control, number of sequences after merging them by their 3’ and resulting non-chimeric ASV.

SampleID	Input	Denoised	Merged	Non-chimeric
Bs1	222461	143007	73638	47964
Bs2	205171	126294	59986	40689
Bs3	208628	137845	72140	47179
Sc1	179903	109927	50973	38816
Sc2	243159	152986	83377	54381
Sc3	186513	123113	69646	46552

Supplementary table S3. Mean relative abundance of fungal phylum identified in *Baccharis scandens* and *Solanum chilense* rizhospheres.

**Taxa [Phylum]**	***Baccharis scandens***	***Solanum chilense***
Ascomycota	82.81 ± 4.69	46.08 ± 3.59
Basidiomycota	2.60 ± 1.40	12.64 ± 2.04
Unid. Chromista	0.27 ± 0.20	0.42 ± 0.33
Mucoromycota	0.30 ± 0.17	0.26 ± 0.01
Mortierellomycota	0.24 ± 0.22	0.23 ± 0.35
Glomeromycota	0.50 ± 0.53	0.00 ± 0.00
Chytridiomycota	0.15 ± 0.08	0.18 ± 0.09

Supplementary table S4. Mean relative abundance 15 most abundant fungal families identified in *Baccharis scandens* and *Solanum chilense* rizhospheres.

Taxa [Family]	*Baccharis scandens*	*Solanum chilense*
Aspergillaceae	26.57 ± 21.19	2.34 ± 0.26
Pleosporaceae	12.98 ± 13.14	6.65 ± 4.02
Nectriaceae	6.34 ± 0.88	9.20 ± 4.79
Saccharomycetaceae	8.57 ± 7.23	4.10 ± 4.24
Dipodascaceae	3.26 ± 1.77	3.61 ± 3.47
Cladosporiaceae	4.33 ± 6.86	0.69 ± 0.84
Psathyrellaceae	0.39 ± 0.27	4.25 ± 0.28
Filobasidiaceae	0.89 ± 0.87	3.40 ± 2.01
Dydimellaceae	2.64 ± 2.06	1.32 ± 1.93
Sporormiaceae	1.86 ± 0.99	1.55 ± 0.69
Heliotales i.s.	3.16 ± 2.93	0.09 ± 0.16
Agaricaceae	0.02 ± 0.02	2.46 ± 2.22
Pyronematacaee	1.95 ± 1.36	0.33 ± 0.22
Ascobolaceae	0.03 ± 0.03	2.08 ± 3.56
Chaetomiaceae	0.54 ± 0.39	1.38 ± 1.44

Supplementary table S5. Mean relative abundance of bacterial phylum identified in *Baccharis scandens* and *Solanum chilense* rizhospheres.

Taxa [Phylum]	*Baccharis scandens*	*Solanum chilense*
Actinobacteria	38.9 ± 3.3	26.9 ± 5.27
Proteobacteria	24.4 ± 0.84	28.4 ± 3.80
Bacteroidetes	5.89 ± 1.4	4.93 ± 1.65
Planctomycetes	2.82 ± 0.29	5.18 ± 3.67
Chloroflexi	4.88 ± 0.57	5.46 ± 2.37
Firmicutes	4.74 ± 1.2	2.36 ± 0.31
Cyanobacteria	1.75 ± 1.52	5.27 ± 3.06
Acidobacteria	1.85 ± 0.55	3.98 ± 2.10
Verrucomicrobia	1.47 ± 0.34	2.41 ± 0.68
Gemmatimonadetes	1.74 ± 0.51	1.73 ± 0.68
TM7	1.53 ± 0.16	0.81 ± 0.17
Nitrospirae	0.17 ± 0.01	0.49 ± 0.23
Armatimonadetes	0.11 ± 0.07	0.31 ± 0.30
Tenericutes	0.33 ± 0.35	0.00 ± 0.00
OD1	0.14 ± 0.06	0.11 ± 0.12

Supplementary table S6. Mean relative abundance 15 most abundant bacterial families identified in *Baccharis scandens* and *Solanum chilense* rizhospheres.

**Taxa [Family]**	***Baccharis scandens***	***Solanum chilense***
Micrococcaceae	14.30 ± 3.17	5.68 ± 3.90
Nocardioidaceae	5.29 ± 1.75	2.51 ± 0.54
Sphingomonadaceae	3.70 ± 0.34	3.46 ± 0.81
Streptomycetaceae	2.06 ± 1.00	3.40 ± 1.67
Rhodospirillaceae	1.59 ± 0.36	3.06 ± 0.46
Geodermatophilaceae	3.23 ± 1.59	1.01 ± 0.20
Chitinophagaceae	1.73 ± 1.20	1.93 ± 0.82
Micromonosporaceae	1.60 ± 0.30	1.72 ± 0.36
Comamonadaceae	1.47 ± 0.43	1.69 ± 1.53
Pirellulaceae	1.10 ± 0.24	1.97 ± 1.80
Cytophagaceae	1.39 ± 0.55	1.28 ± 1.02
Gaiellaceae	0.58 ± 0.21	1.68 ± 1.53
Planococcaceae	1.34 ± 0.56	0.86 ± 0.27
Bacillaceae	1.44 ± 0.44	0.68 ± 0.26
Phyllobacteriaceae	0.87 ± 0.26	1.23 ± 0.51

Supplementary table S7. Alpha diversity of rhizosphere fungi. Three measures are shown: Shannon diversity index, observed number of OTUs per sample and Pielou's Evenness.

SampleID	Rizhosphere	Shannon		Observed OTU		Evenness	
Bs1	*Baccharis scandens*	5.8084		645		0.6223	
Bs2	*Baccharis scandens*	4.8428	5.48 ± 0.55	635	595.33 ± 77.53	0.5201	0.5964 ± 0.0672
Bs3	*Baccharis scandens*	5.8095		506		0.6467	
Sc1	*Solanum chilense*	5.0363		400		0.5826	
Sc2	*Solanum chilense*	5.6896	5.40 ± 0.33	754	541.67 ± 187.28	0.5952	0.5976 ± 0.0163
Sc3	*Solanum chilense*	5.4598		471		0.6149	

Supplementary table S8. Alpha diversity of rhizosphere bacteria. Three measures are shown: Shannon diversity index, observed number of OTUs per sample and Pielou's Evenness.

SampleID	Rizhosphere	Shannon		Observed OTU		Evenness	
Bs1	*Baccharis scandens*	9.4541		1299		0.9140	
Bs2	*Baccharis scandens*	9.4260	9.44 ± 0.01	1182	1265 ± 72.95	0.9235	0.9160 ± 0.0068
Bs3	*Baccharis scandens*	9.4339		1316		0.9104	
Sc1	*Solanum chilense*	9.6225		1134		0.9483	
Sc2	*Solanum chilense*	9.8587	9.58 ± 0.30	1548	1293 ± 222.86	0.9304	0.9281 ± 0.0215
Sc3	*Solanum chilense*	9.2596		1198		0.9055	

Supplementary table S9. Relative abundance (%) of *Phylum* that were significantly abundant in one rizhosphere, and their respective LDA scores. Higher values are highlighted (bolder). Relative abundance (%) of *genus* that are significantly more abundant in on rizhosphere and their respective LDA score. Higher values are highlighted (bolder).

**A**	**Taxa [phylum]**	***Baccharis scandens***	***Solanum chilense***	**LDA Score**
	Glomeromycota	**0.50 ± 0.53**	0.00 ± 0.00	3.46
	Ascomycota	**82.81 ± 4.69**	46.08 ± 3.59	5.26
	Basidiomycota	2.60 ± 1.40	**12.64 ± 2.04**	4.71
**B**	**Taxa [genus]**	***Baccharis scandens***	***Solanum chilense***	**LDA Score**
	Funneliformis	**0.072 ± 0.080**	0.000 ± 0.000	2.79
	Spizellomyces	**0.117 ± 0.087**	0.004 ± 0.007	2.89
	Scutellinia	**0.074 ± 0.037**	0.019 ± 0.018	3.11
	Paraphoma	**0.682 ± 0.478**	0.050 ± 0.008	3.50
	Diversispora	**0.117 ± 0.087**	0.004 ± 0.007	3.53
	Tricharina	**1.204 ± 0.743**	0.036 ± 0.032	3.72
	Cadophora	**3.164 ± 2.937**	0.076 ± 0.132	4.14
	Monosporascus	**0.002 ± 0.000**	0.000 ± 0.000	4.61
	Penicillium	**26.315 ± 21.135**	1.235 ± 0.951	5.16
	Oidiodendron	0.001 ± 0.002	**0.007 ± 0.002**	3.88
	Talaromyces	0.000 ± 0.000	**0.003 ± 0.002**	3.10

Supplementary table S10. Relative abundance (%) of *Phylum* that were significantly abundant in one rizhosphere, and their respective LDA scores. Higher values are highlighted (bolder). Relative abundance (%) of *genus* that are significantly more abundant in on rizhosphere and their respective LDA score. Higher values are highlighted (bolder).

**A**	**Taxa [phylum]**	***Baccharis scandens***	***Solanum chilense***	**LDA Score**
	Actinobacteria	**38.9 ± 3.30**	26.9 ± 5.27	4.75
	Firmicutes	**4.74 ± 1.20**	2.36 ± 0.31	4.02
	TM7	**1.53 ± 0.16**	0.81 ± 0.17	3.61
	Tenericutes	**0.33 ± 0.35**	0.00 ± 0.00	3.23
	Nitrospirae	0.17 ± 0.01	**0.49 ± 0.23**	3.25

**B**	**Taxa [genus]**	***Baccharis scandens***	***Solanum chilense***	**LDA Score**
	Porifericola	**0.039 ± 0.037**	0.000 ± 0.000	2.60
	Georgfuchsia	**0.152 ± 0.048**	0.001 ± 0.001	2.99
	Nodularia	**0.082 ± 0.032**	0.000 ± 0.000	2.99
	Nostoc	**0.253 ± 0.209**	0.000 ± 0.000	3.13
	Sporichthya	**0.179 ± 0.036**	0.017 ± 0.029	2.97
	Kineosporia	**0.137 ± 0.045**	0.046 ± 0.044	2.74
	Methylotenera	**0.043 ± 0.016**	0.000 ± 0.000	2.77
	Lentzea	**0.173 ± 0.069**	0.059 ± 0.063	2.83
	Oscillochloris	**0.069 ± 0.027**	0.009 ± 0.016	3.06
	Haloferula	**0.079 ± 0.075**	0.000 ± 0.000	2.78
	Rhodocytophaga	**0.234 ± 0.119**	0.065 ± 0.058	2.95
	Bacillus	**0.631 ± 0.107**	0.369 ± 0.109	3.08
	Asteroleplasma	**0.325 ± 0.348**	0.002 ± 0.004	3.23
	Rhizobium	**0.602 ± 0.324**	0.093 ± 0.116	3.39
	Blastococcus	**2.757 ± 1.191**	0.866 ± 0.203	4.01
	Arthrobacter	**14.17 ± 2.973**	5.558 ± 3.693	4.67
	Methylovorus	0.000 ± 0.000	**0.121 ± 0.136**	2.87
	Nitrospira	0.166 ± 0.013	**0.486 ± 0.228**	3.25
	Dongia	0.017 ± 0.030	**0.120 ± 0.035**	2.79
	Reyranella	0.057 ± 0.051	**0.325 ± 0.081**	3.15
	Lactobacillus	0.000 ± 0.000	**0.018 ± 0.012**	3.01
	Fimbriimonas	0.000 ± 0.000	**0.091 ± 0.057**	2.99
	Rhodopila	0.000 ± 0.000	**0.130 ± 0.114**	2.85

Supplementary table S11. Relative abundance (%) of functional annotations by plant hosts.

**Annotation**	***Baccharis scandens***	***Solanum chilense***
Chemoheterotrophy	36.66 ± 4.16	34.51 ± 2.12
Aerobic chemoheterotrophy	35.36 ± 4.09	31.72 ± 3.11
Aromatic compound degradation	7.19 ± 3.39	4.00 ± 1.12
Chloroplasts	0.45 ± 0.52	10.17 ± 4.83
Phototrophy	2.92 ± 2.10	1.43 ± 0.21
Photoautotrophy	2.87 ± 2.16	0.88 ± 0.22
Cyanobacteria	2.86 ± 2.17	0.80 ± 0.32
Oxygenic photoautotrophy	2.86 ± 2.17	0.80 ± 0.32
Ureolysis	1.67 ± 1.41	1.93 ± 1.71
Nitrification	0.49 ± 0.10	1.65 ± 1.09
Intracellular parasites	0.41 ± 0.29	1.47 ± 0.34
Nitrogen fixation	1.06 ± 0.48	0.77 ± 0.42
Hydrocarbon degradation	1.00 ± 0.90	0.79 ± 0.16
Chitinolysis	0.53 ± 0.26	0.98 ± 0.59
Aerobic nitrite oxidation	0.32 ± 0.02	1.17 ± 0.82

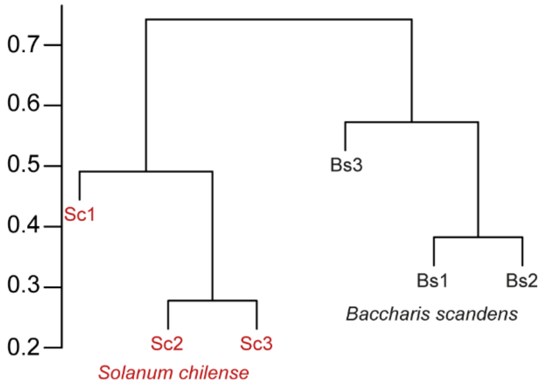

Supplementary Figure S1. Hierarchical clustering analysis of fungal communities.

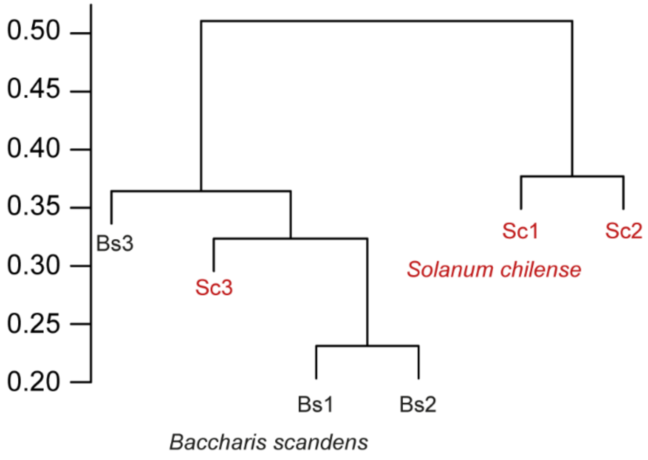

Supplementary Figure S2. Hierarchical clustering analysis of bacterial communities.
